# Programmed death one homolog maintains the pool size of regulatory T cells by promoting their differentiation and stability

**DOI:** 10.1038/s41598-017-06410-w

**Published:** 2017-07-20

**Authors:** Qi Wang, Jianwei He, Dallas B. Flies, Liqun Luo, Lieping Chen

**Affiliations:** 1 Laboratory of Immunotherapy, Sun Yat-Sen University, Guangzhou, Guangdong P.R. China; 2 Department of Immunobiology, Yale University, New Haven, Connecticut USA

**Keywords:** Immunology, Inflammation

## Abstract

Programmed death one homolog (PD-1H) is an immunoglobulin superfamily molecule and primarily acts as a coinhibitor in the initiation of T cell response to antigens. Here, we report that genetic ablation of PD-1H in mice blocks the differentiation of naive T cells to Foxp3^+^ inducible Treg cells (iTreg) with a significant decrease of iTreg in lymphoid organs. This effect of PD-1H is highly specific for iTreg because both naturally generated iTreg in gut-related tissues and *in vitro* induced iTreg by TGF-β were decreased whereas the genesis of natural Treg (nTreg) remains normal. The suppressive function of both iTreg and nTreg, however, is not affected by the loss of PD-1H. In addition to decreased production, PD-1H deficient iTreg could also rapidly convert to CD4^+^ T helper 1 or T helper 17 cells in an inflammatory environment. Our results indicate that PD-1H is required for maintenance of iTreg pool size by promoting its differentiation and preventing its conversion to other CD4^+^ T cell subsets. These findings may have important implications for manipulating Tregs to control inflammation.

## Introduction

Regulatory T cells (Treg) are a subset of CD4^+^ T cells with broad functions from maintenance of self-tolerance to regulation of a magnitude of immune responses^1–3^. Treg are not terminally differentiated and can be converted to other CD4^+^ T cell subsets including Th1 and Th17 during inflammation^4, 5^. It has been shown that the transcription factor Foxp3 plays an essential role in the establishment of a functional and committed regulatory T cell lineage. Foxp3^+^ Treg cells can be divided into thymus-derived natural Treg cells (nTreg) and inducible Treg cells (iTreg) by TGF-β^6, 7^, which regulate the differentiation of iTreg cells and stabilization of thymus-derived nTreg^8–11^. In the periphery, the differentiation of iTreg cells is largely driven by the microenvironment. For example, inflammatory cytokines IFN-γ and IL-4 inhibit TGF-β-induced iTreg cells, while IL-6 directs Th17 cell differentiation in the presence of TGF-β^12–14^. The plasticity of Treg cells may thus determine the direction of an ongoing immune response and control inflammation as shown in several mouse models including models of colitis, acute graft versus host diseases (GVHD), and asthma^15^.

PD-1H (also called Gi24, Dies1, B7-H5, VISTA and DD1α) is a cell surface immunoglobulin superfamily molecule with immune modulatory functions in addition to its myriad of roles regulating the differentiation of osteoblast, adipocyte, and embryonic stem cells^16–21^ and cell apoptosis^22^. PD-1H is constitutively expressed on hematopoietic cells, such as T cells, NK cells, monocytes, and DCs, but not on B cells^17, 21, 23^. Unlike CTLA-4 knockout (KO) mice that rapidly develop lymphoproliferative phenotypes and fatal systemic autoimmune diseases^24^, PD-1H deficiency has a much more mild phenotype: young PD-1H KO mice have normal numbers of T cells, NK cells, B cells, macrophages, and monocytes, while older mice experience spontaneous T cell activation, and increased levels of memory cells and larger spleen size^25, 26^. Furthermore, PD-1H deficient mice were more susceptible to acute inflammation and immune response to antigens as shown in accelerated Con A-induced acute hepatitis and GVHD^26^. PD-1H has been shown to function on professional antigen-presenting cells (APCs) and T cells as either a ligand or a receptor, respectively, in several *in vitro* and *in vivo* studies^25–27^. Consistent with these findings, agonistic mAb to PD-1H have proven to be immune inhibitors for various types of immune responses to antigens^26^, whereas antagonistic mAb were shown to be immune stimulators^28, 29^. Although the counter-receptor(s) of PD-1H have yet to be identified, a recent study indicated that PD-1H/DD1α could mediate its effect via a hemophilic interaction^22^.

Our early studies show that PD-1H is constitutively expressed on Treg^23^ and several subsequent studies implicate its role in the regulation of Treg functions. PD-1HIg fusion protein promoted the induction of Foxp3^+^ iTreg in the presence of TGF-β in both mice and human CD4^+^ T cells *in vitro*^28, 29^. Infusion of a PD-1H mAb in the B16-OVA tumour model reduced the differentiation of tumour antigen-specific iTreg cells. This result was interpreted as a blockade of the PD-1H interaction with its putative counter-receptor by this mAb^28^. A different PD-1H agonist mAb MH5A, however, was shown to promote TGF-β induced Treg cells *in vitro*, and infusion of MH5A suppressed progression of GVHD in mouse models, accompanied by expansion of iTreg^30^. While these data suggest a possible role for PD-1H in Treg induction and function, it has yet to be elucidated whether PD-1H has a direct effect on Treg cells. More importantly, the mechanisms underlying the modulatory effect of Treg cells by PD-1H are unknown. In this study, we show that PD-1H is a critical factor for the differentiation and stability of iTreg cells.

## Results

### PD-1H is required for *de novo* induction of Treg cells

**Figure 1:**
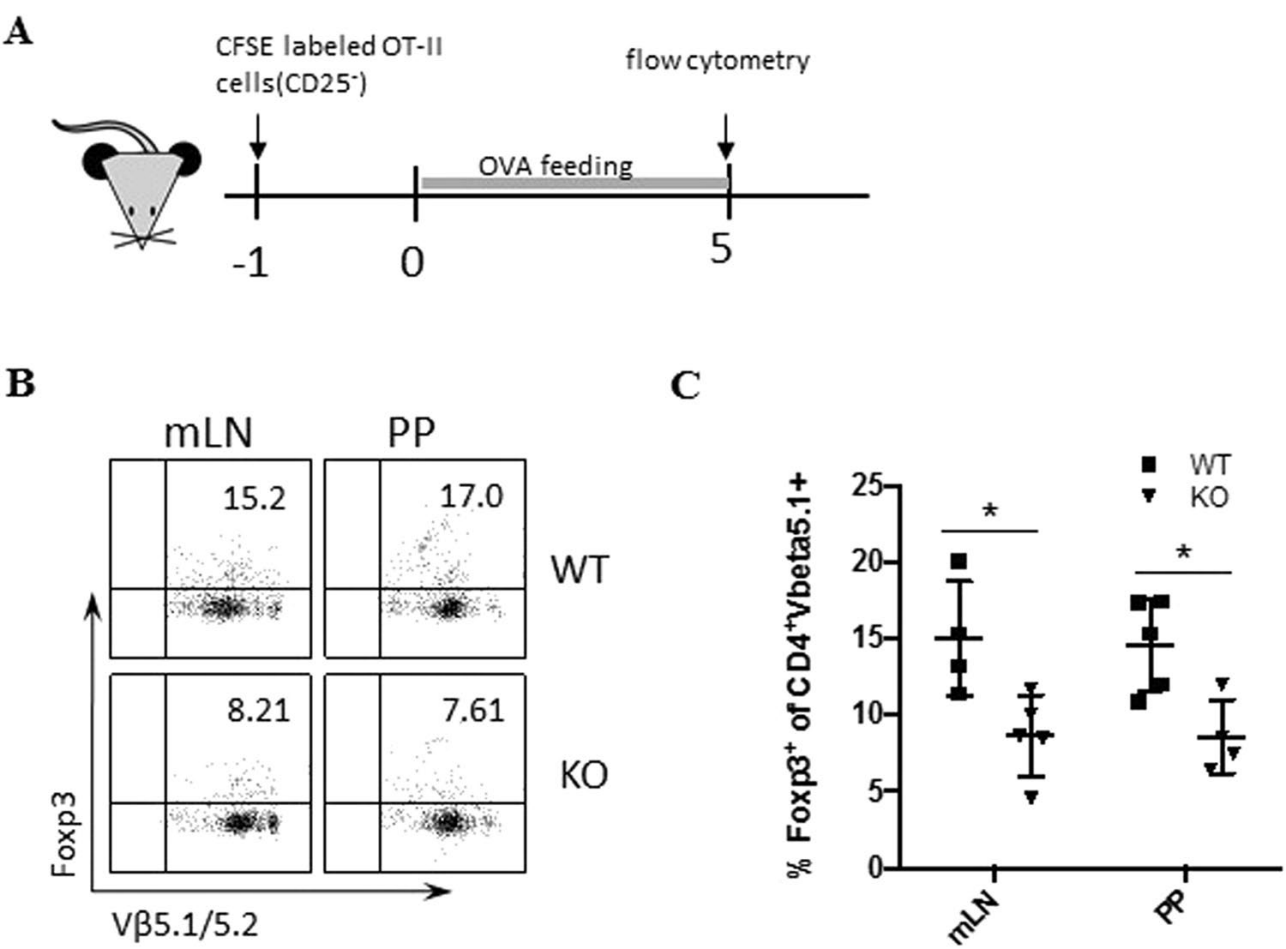
Effect of PD-1H in *de novo* generation of Foxp3^+^ iTreg cells. (**A**) Naïve T cells purified from WT OT-II or PD-1H KO OT-II mice were first labelled with 5 μM CFSE and subsequently transferred i.v. to B6 mice at 2 × 10^6^/mouse. Mice were fed with 1.5% OVA in the drinking water 24 hours later for 5 days. Foxp3 frequency on the gated CD4^+^CFSE^+^Vβ5.1/5.2 TCR^+^ was analysed by flow cytometry in the representative mice. (**B**,**C**) Summary of an experiment with each symbol representing an individual mouse (n = 5). Data shown are representative of 3 independent experiments.

We first explored the role of PD-1H in an oral tolerance model where oral feeding of chicken ovalbumin (OVA) is shown to promote expansion and *de no*vo generation of iTreg cells in the periphery^31–33^. OT-II TCR transgenic mice were backcrossed to PD-1H KO mice to generate a new KO OT-II strain^26^. CD4^+^ T cells were purified from spleen cells of WT OT-II or KO OT-II mice were depleted of CD25^+^ T cells (to avoid nTreg contamination) and subsequently transferred into WT B6 mice. Mice were then fed with 1.5% OVA in their drinking water for 5 consecutive days (Fig. 1A). Consistent with previously published findings, Foxp3^+^Vβ5.1/5.2^+^ OT-II cells could be detected in gut-related lymphoid organs including mesenteric lymph nodes (mLN) and Peyer’s patch (PP) while fewer cells were detected in the spleen, peripheral LN, and lamina propria (LP) (data not shown), indicating a Treg response in gut-related lymphoid organs to OVA. In the absence of PD-1H, Foxp3^+^Vβ5.1/5.2^+^ OT-II cells were significantly decreased in the mLN and PP compared with WT OT-II (Fig. 1B and C), although division rates in WT and KO OT-II cells in both mLN and PP were comparable based on the CFSE dilution (Supplementary Fig. S1A). Similar results were observed when naive WT OT-II cells and KO OT-II cells were co-transferred into OVA feeding mice (Supplementary Fig. S1B and C). We found that in the same host mice, PD-1H deficiency on OT-II cells led to impaired differentiation of Foxp3^+^ T cells compared with co-transferred WT OT-II cells (Supplementary Fig. S1B and C). Our results indicate that the loss of PD-1H impairs the induction of OVA-specific iTreg cells *in vivo*.

Homeostatic proliferation upon transferring naïve CD4^+^ T cells into the lymphopenic mice could upregulate Foxp3 expression and these Foxp3^+^ iTreg cells acquired a suppressive function *in vitro* ^34^. To determine whether PD-1H affects this process, naïve CD4^+^ T cells from WT (CD45.1) and KO (CD45.2) mice were mixed at a 1:1 ratio, and transferred into Rag1 KO mice. The percentage and absolute number of CD25^+^Foxp3^+^ T cells in the indicated organs were analysed 3 weeks later (Supplementary Fig. S2). We found that the total numbers of KO CD4^+^ T cells increased by more than twice the number of WT CD4^+^ T cells in the spleen (data not shown). However, a significantly lower frequency of CD25^+^Foxp3^+^ iTreg cells were found in the spleen, LN, and mLN within the KO CD4^+^ T cells compared with WT CD4^+^ T cells. In addition, PD-1H loss on CD4^+^ T cells also led to a decreased number of Foxp3^+^ iTreg cells compared with WT CD4^+^ T cells both in the LN and mLN, although an insignificant change was observed in the spleen (Supplementary Fig. S2B). Besides, we found that the recovered KO CD4^+^ T cells produced significantly higher IFN-γ and IL-17 cytokines compared with WT CD4^+^ T cells in the lymphopenic environment (Supplementary Fig. S2C). Therefore, we concluded that PD-1H is required for *de novo* generation of Foxp3^+^ iTreg cells.

### PD-1H is required for expansion, but not generation and function, of iTreg cells

PD-1H KO mice display normal numbers of nTreg cells in the thymus, spleen, and lymph nodes^23, 26^. In addition, the phenotype and suppressive function of nTreg cells in PD-1H KO mice was also comparable to those in WT littermates (Supplementary Fig. S3). These findings are consistent with previous observations that young PD-1H KO mice had no obvious autoimmune-like phenotypes^21, 26^. Therefore, PD-1H does not seem to be required for the development and functional maturation of nTreg in lymphoid organs.

**Figure 2:**
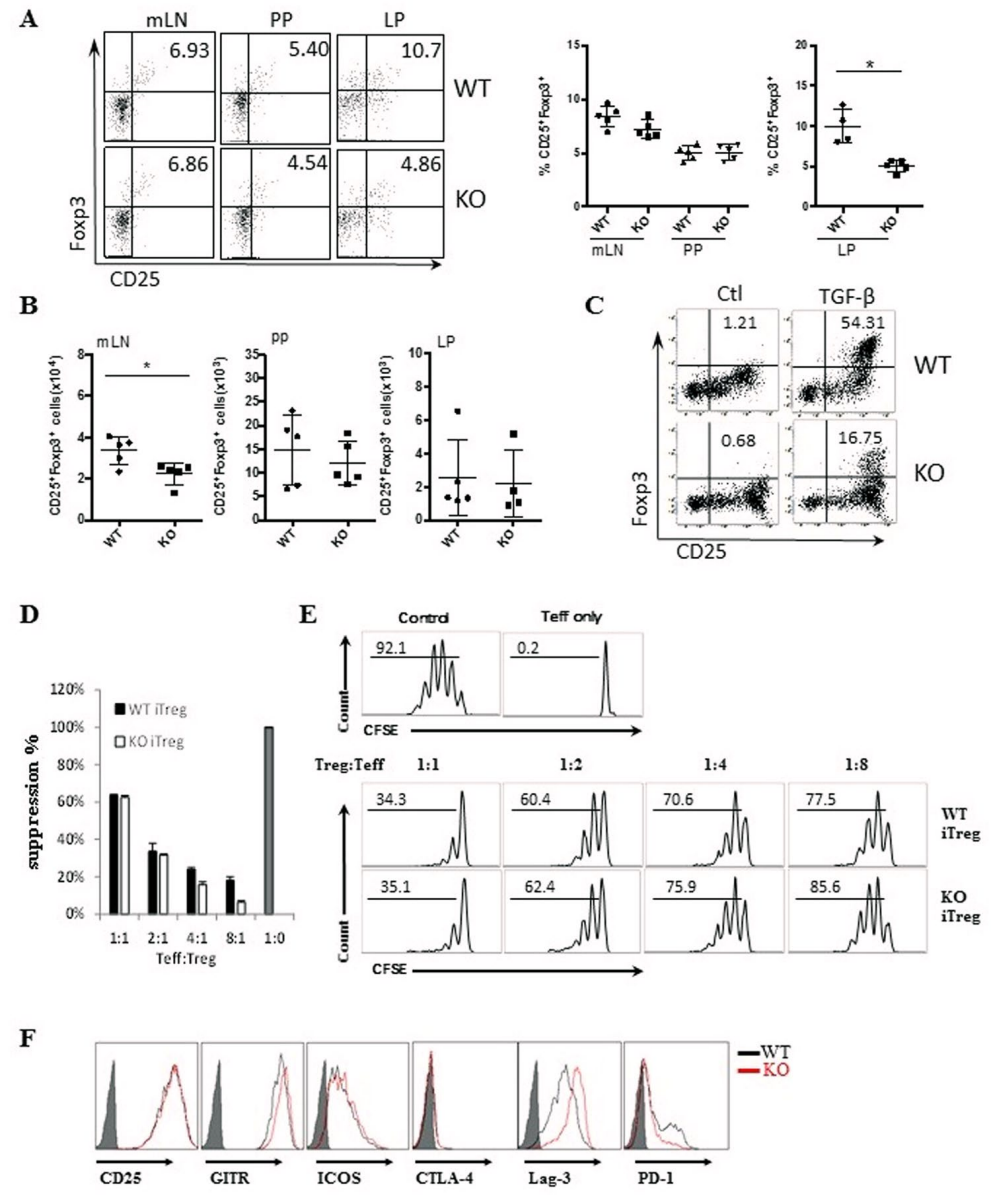
Effect of PD-1H on the conversion and function of iTreg. (**A**) PD-1H deficiency in the natural development of iTreg in gut-associated lymphoid organs. The percentages of CD25^+^Foxp3^+^ Treg cells in the mesenteric lymph nodes (mLN), payer’s patch (PP) and lamina propria (LP) were determined by cell surface CD25 and intracellular Foxp3 expression with specific antibodies in flow cytometry. PD-1H KO mice and their WT littermates (n = 5 per group) were used for analysis. The left panel represents paired individual mice from each group and the right panel is a summary of one representative experiment (n = 5 in each group). (**B**) Absolute numbers of CD25^+^Foxp3^+^ Treg in gut-associated lymphoid organs. (**C**) *In vitro* induction of iTreg. CD4^+^CD25^−^CD62L^hi^ naïve T cells from WT and PD-1H KO mice were stimulated with anti-CD3/CD28 in the presence or absence of 5 ng/ml TGF-β for 3–5 days. The frequency of CD25^+^Foxp3^+^cells was determined by intracellular staining. (**D**,**E**) Assessing the suppressive function of iTreg *in vitro*. Naïve CD4^+^ T cells from WT or PD-1H KO Foxp3 (GFP) mice were induced to become Foxp3 (GFP^+^) iTreg cells as described above. Naïve CD8^+^ T cells or CD4^+^ T cells were purified from B6 mice, labelled with CFSE, and co-cultured with sorted GFP^+^ iTreg cells in the presence of anti-CD3 at the indicated Treg/Teff cells ratio. The decrease of CSFE upon inclusion of iTreg cells was determined by comparison with the wells without the addition of iTreg cells. Data shown are representative of at least 3 independent experiments. Teff only: T cells without anti-CD3 stimulation; Control: Teff cells with anti-CD3 without the inclusion of iTreg cells. (**F**) The expression of CD25, GITR, Lag-3, CTLA-4, ICOS and PD-1 on the iTreg from WT or PD-1H KO Foxp3(GFP) mice were determined by flow cytometry gating on the CD4^+^Foxp3(GFP^+^) cells.

Because iTreg cells are generated mainly in the gut under either a steady state or inflammation^31, 33^, we next examined whether the lack of PD-1H affects iTreg generation and function. Although a similar proportion of Foxp3^+^ Treg cells in the mLN and PP has been found in both PD-1H KO mice and WT littermates, Foxp3^+^ cells in the LP of PD-1H KO mice were significantly lower, although the absolute number of Foxp3^+^ cells was unchanged in the absence of PD-1H (Fig. 2A and B). Similar results were also found in the LP when the PD-1H KO/WT mice were backcrossed to Foxp3 (GFP) mice in which the GFP gene was under the control of the Foxp3 promoter (data not shown). These data suggest a defect in the differentiation of iTreg cells in the absence of PD-1H *in vivo*. To further validate our findings in the PD-1H KO mice, mixed bone marrow chimera was generated. Sub-lethally irradiated B6 mice were adoptively transferred with mixed bone marrow from WT (CD45.1) and KO mice (CD45.2). The frequency of Foxp3^+^ cells in the indicated organs were analysed after 10 weeks. We found that the total number of KO T cells increased nearly two fold compared to WT T cells (Supplementary Fig. S4A). In contrast, the levels of KO Foxp3^+^ T cells were significantly lower than both WT T cells and recipient T cells in the peripheral organs (Supplementary Fig. S4B and C). In the spleen of chimeric mice, the absolute numbers of KO Foxp3^+^ T cells were also lower than WT T cells whereas the Foxp3^−^ effector T cells (Teff) expanded much more vigorously (Supplementary Fig. S4C). These data indicate that PD-1H is co-inhibitory for the activation and homeostasis of T cells but promotes the development of Foxp3^+^ Treg cells *in vivo*.

We next tested if the loss of PD-1H could affect the conversion of iTreg cells *in vitro*. Naïve CD4^+^CD25^−^CD62L^hi^ cells were purified from WT and PD-1H KO spleen cells, and stimulated with anti-CD3/CD28 in the presence of TGF-β. The frequency of Foxp3^+^ iTreg cells that were differentiated from WT naive T cells were significantly higher than that from the KO naive T cells (Fig. 2C). Taken together, we conclude that PD-1H is required for *de novo* differentiation of iTreg.

To further test if PD-1H loss affects iTreg function, iTreg cells from WT or PD-1H KO Foxp3 (GFP) mice were induced *in vitro* as described above and purified by sorting. Teff were generated by anti-CD3 stimulation of purified CD4^+^CD25^−^CD62L^hi^ naïve T cells. WT or KO iTreg cells were co-cultured with effector cells at the indicated Treg/Teff ratios in the presence of irradiated spleen cells, as antigen-presenting cells plus soluble anti-CD3 for 3 days. Teff were CFSE-labelled before co-culture and decreased CFSE dilutions were used as an indication of Treg suppression. As shown in Fig. 2D and E, iTreg cells from both WT and KO mice suppressed proliferation of Teff with comparable activity. Consistent with these findings, we did not find significant differences in the expression of activation markers CD25, GITR, Lag-3, CTLA-4, ICOS, or PD-1 in WT vs. KO iTreg (Fig. 2F). Therefore, PD-1H does not affect the suppressive function of iTreg despite its effect on the differentiation of iTreg.

Finally, we tested the effect of a PD-1H agonistic mAb mam82 on iTreg expansion^30^. Naïve T cells were sorted from WT Foxp3 (GFP) knock-in mice and subsequently stimulated with anti-CD3/CD28 in the presence of TGF-β. Induction of GFP^+^CD25^+^ iTreg cells was measured upon stimulation. Inclusion of mam82 slightly increased Foxp3^+^CD25^+^ iTreg expansion, albeit the effect appeared moderate (Supplementary Fig. S5A and B). This moderate effect, however, may be due to a rapid loss of cell surface PD-1H on T cells *in vitro*^35^. Collectively, our data indicate that PD-1H is required for the generation of iTreg from naïve T cells, but does not affect their suppressive functions.

### PD-1H regulates iTreg differentiation via cytokines

**Figure 3:**
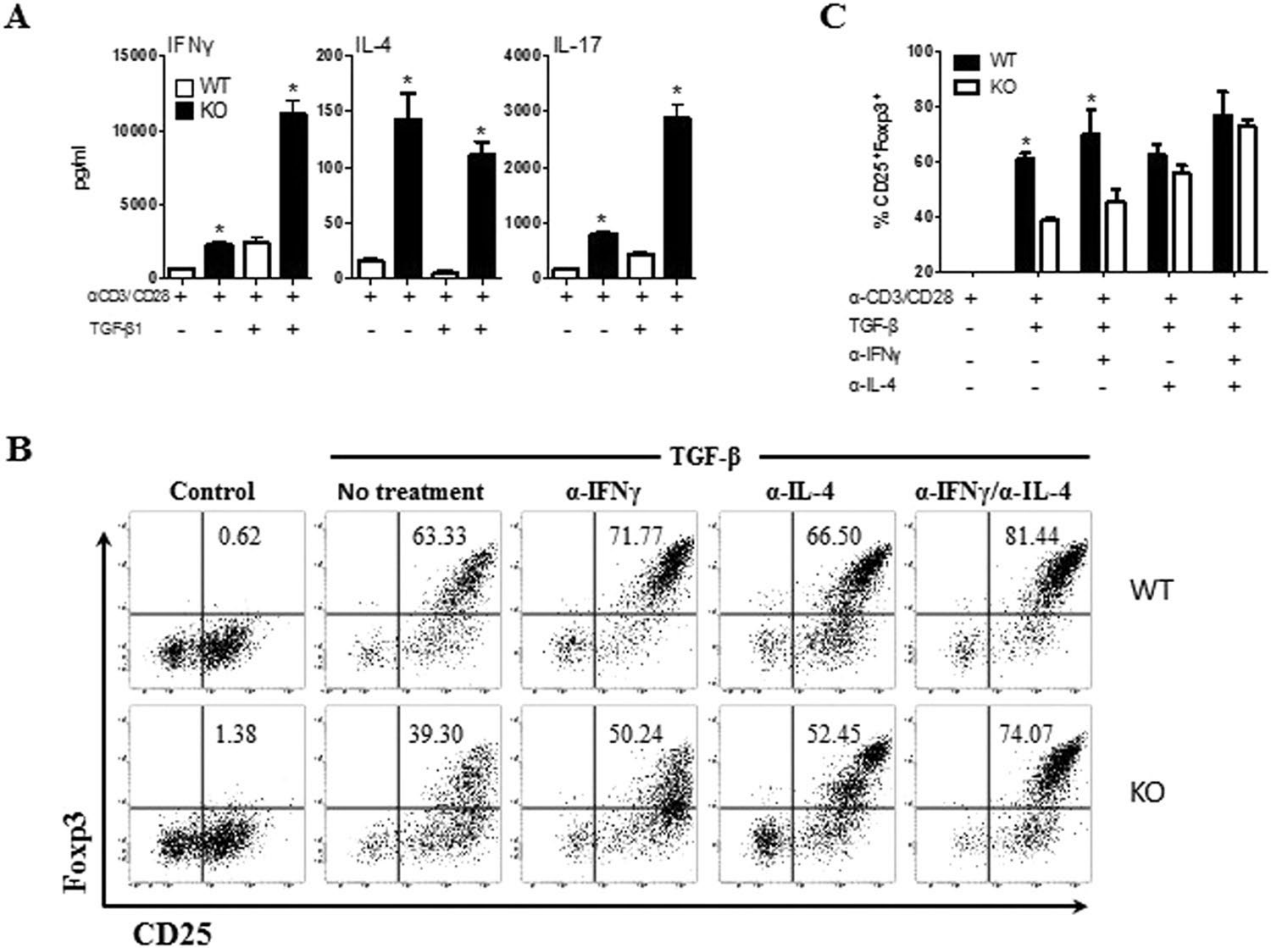
Effect of the cytokine milieu on the PD-1H-mediated defects on the conversion of iTreg cells. (**A**) Cytokine profile in the induction of iTreg cells in the absence of PD-1H. Naïve T cells were induced to iTreg *in vitro* as described above and the cultured supernatants were collected at day 4 to determine cytokine levels by mouse Th1/TH2/Th17 CBA kits. (**B**) Neutralizing mAb to IFN-γ and IL4 were added to the culture at the beginning of the culture to induce iTreg cells from WT or PD-1H KO naïve T cells *in vitro*. CD25^+^Foxp3^+^ cells were assessed 3–5 days after the culture. The presented results were from a pair of mice. (**C**) Histogram presentation of the data from (**B**) are from a group of 5 mice. Data shown are representative of at least 3 independent experiments.

Previously, we showed that activated PD-1H KO CD4 T cells *in vitro* produced higher levels of IFN-γ and IL-17 than WT T cells^26^. Furthermore, co-transfer of naïve T cells from WT and KO mice into lymphopenic mice results in decreased Foxp3^+^ T cells and increased IFNγ^+^ and IL-17^+^ cells in KO versus WT T cells (Supplementary Fig. S2). Therefore, cytokines may modulate the differentiation of iTreg cells in the absence of PD-1H. To test this, purified CD4^+^ T cells were first stimulated with anti-CD3/CD28 and cultured supernatants were collected for cytokine detection. Consistent with our previous findings, activated KO CD4^+^ T cells produced higher levels of IFN-γ and IL-17 compared with WT CD4^+^ T cells (Fig. 3A). In the presence of TGF-β, production of these cytokines was further increased, while there was no change in TNF-α (data not shown). In addition, PD-1H KO CD4^+^ T cells produced more IL-4 with or without TGF-β (Fig. 3A). Because these cytokines are produced by different subsets of CD4^+^ T cells, our findings support PD-1H as a pan inhibitor of T helper cells.

Neutralizing mAb to IL-4 and/or IFN-γ were added to the cultures to exploit the effect of these cytokines in the induction of iTreg cells. Neutralizing either IL-4 or IFN-γ partially restores differentiation of PD-1H KO iTreg cells compared to WT control, while inclusion of both mAbs restores the majority of the activity. As the controls, IFN-γ/IL-4 neutralizing mAbs, either used alone or in combination, could also enhance iTreg differentiation (Fig. 3B and C). These findings indicate that impaired iTreg differentiation in the absence of PD-1H is due, at least in part, to altered cytokine production by T cells.

### Loss of PD-1H facilitates the conversion of iTreg to Th17 in an inflammatory environment

**Figure 4:**
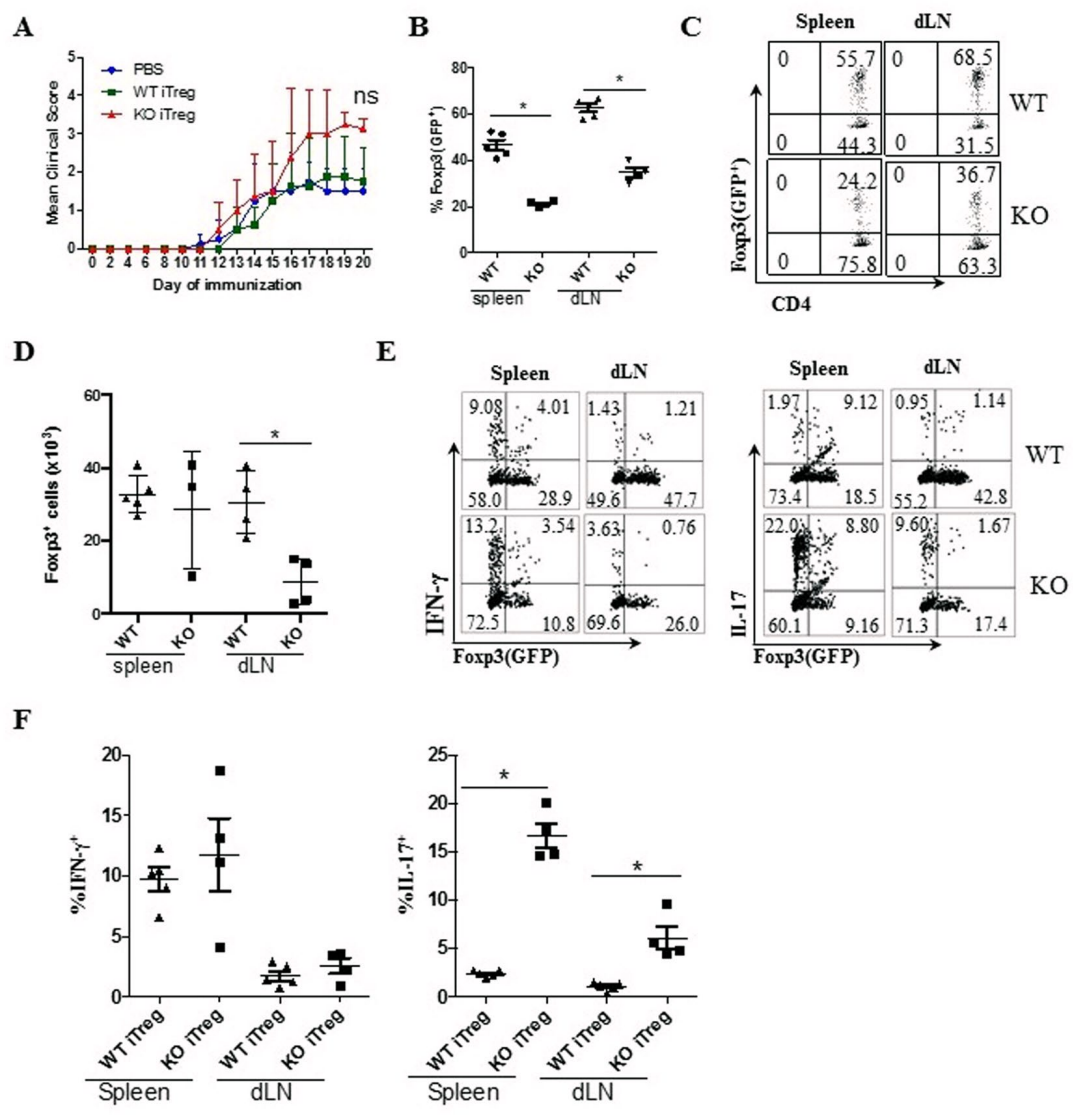
Effect of PD-1H on the stability of iTreg cells in the EAE model. (**A**) CD45.2^+^Foxp3(GFP^+^) iTreg cells from WT or KO were obtained by cell sorting after induction *in vitro*. 1 × 10^6^ WT or PD-1H KO Foxp3 (GFP^+^) iTreg cells were transferred i.v. into the CD45.1 B6 mice (n = 4 or 5 per group) before the immunization with MOG_35–55_ peptide. Control mice were inoculated with PBS. EAE disease progression and severity was monitored as clinical score (see Methods). *P < 0.05, (two-way ABOVA test). Data shown represent 1 of 3 experiments with similar results and disease phenotypes. (**B**,**C**) The spleen and draining LN (dLN) cells at Day 13 of EAE induction were gated on CD45.2^+^CD4^+^ and analysed for Foxp3 (GFP^+^) iTreg cells. (**D**) Absolute numbers of CD45.2^+^Foxp3^+^ in the spleen and dLN from the EAE mice were counted. (**E**) The spleen and dLN cells from EAE mice at day 13 were re-stimulated *ex vivo* using PMA/Ionomycin/BFA for 4 hours. Cells were gated on CD4^+^CD45.2^+^GFP^+^ for analysis of IFN-γ^+^ or IL-17^+^ expression using intracellular staining. The data from a representative pair of mice are shown. (**F**) Graphic presentation of data in (**E**) shown in a group of 4 or 5 mice. Data shown are representative of at least 3 independent experiments.

Next, we determined whether PD-1H has a direct effect on already differentiated iTreg cells. A murine experimental autoimmune encephalomyelitis (EAE) model was used to test the stability of Foxp3^+^ iTreg cells. Briefly, WT or PD-1H KO Foxp3 (GFP^+^) iTreg cells were generated as described above and sorted by flow cytometry based on GFP positivity (>97% Foxp3^+^, Supplementary Fig. S6A). Foxp3 (GFP^+^) CD45.2^+^ iTreg cells at a concentration of 1 × 10^6^/mouse were transferred intravenously into CD45.1^+^ B6 mice before immunization, where the number of transferred WT iTreg is not sufficient to prevent EAE progression. Mice were then immunized with myelin basic protein to induce EAE, as previously described. In this setting, transfer of WT iTreg slightly delayed the onset of disease compared with the control. However, the transfer of PD-1H KO iTreg cells led to more serious disease, as indicated by clinical score, although no significant difference was found during the peak of disease (Fig. 4A). Interestingly, H&E staining of the spines in EAE mice showed only minor differences of lymphocyte infiltration between KO Treg-transferred mice and WT Treg-transferred mice (data not shown). Then, we analysed the stability of Foxp3 expression in transferred Treg cells during disease progression. Compared with iTreg from WT mice, the Foxp3 frequency (GFP^+^) of KO iTreg was reduced by more than 50% in both spleen and dLN (Fig. 4B and C). Besides, the absolute number of KO Foxp3^+^ cells in the dLN was significantly lower compared with WT Foxp3^+^ cells in the dLN, although the numbers of Foxp3^+^ T cells in the spleen were comparable (Fig. 4C). Thus, our results suggest a role for PD-1H in the maintenance of iTreg phenotype and function.

In the context of convertibility of Treg to other CD4^+^ T cell subsets, our findings suggest a conversion of iTreg to effector T cell subsets, including Th1 and Th17 that are implicated as pathogenic in EAE. To exploit this possibility, spleen and dLN cells from host mice at day 13 were stimulated with PMA/Ionomycin for 4 hours and CD45.2^+^CD4^+^ gated cells were examined for GFP (indication of Foxp3 expression), IFN-γ, and IL-17 using intracellular staining. A moderate increase in the ratio of converted Th1-like cells (IFN-γ^+^Foxp3^−^) was observed in the spleen and dLN of mice transferred with PD-1H KO iTreg cells compared with those transferred with WT iTreg cells (Fig. 4E). Nevertheless, there was no significant difference in the absolute number of converted Th1-like IFN-γ^+^ cells in these two groups of mice (Fig. 4E), suggesting that the conversion of iTreg to Th1 is minimal. However, a large portion of PD-1H KO iTreg cells (16.7% in the spleen and 6.1% in the dLN) were converted into IL-17^+^ cells and there were minimal conversions of IL-17^+^ cells from WT iTreg cells (less than 2% in both spleen and dLN) (Fig. 4E and F). Using the same strategy, we analysed the recipient CD4^+^ T cells (gated on the CD45.1^+^), which showed comparable levels of IFN-γ^+^ and IL-17^+^ cells in both groups of mice transferred with either WT or PD-1H KO iTreg cells (Supplementary Fig. S6B and C). These results indicate that loss of PD-1H facilitates conversion of iTreg to Th17-like cells in the inflammatory environment. Therefore, the effect of PD-1H KO iTreg cells partially facilitating EAE progression (Fig. 4A) could be a result of increased conversion of iTreg to Th17 in an inflammatory environment.

We also evaluated the effect of PD-1H KO Treg on a T-cell transfer model of chronic colitis^36, 37^. The WT and KO Foxp3 (GFP^+^) Treg (CD45.2) were prepared as described above, mixed with congenic CD45.1 Teff (CD45RB^hi^CD25^−^CD4^+^) respectively, and subsequently transferred into Rag1 KO mice. In this experimental setting, we did not observe obvious weight loss during disease progression, even when transferring CD45RB^hi^ Teff alone (Supplementary Fig. S7A). However, co-transfer of PD-1H KO Treg/Teff led to massive leukocyte infiltration and severe tissue damage in the colon by H&E staining, while co-transfer of WT Treg/Teff showed no obvious damage to colon tissue (Supplementary Fig. S7B and C). To determine whether the attenuated suppressive ability of PD-1H KO Treg cells is associated with loss of Foxp3 expression, we assessed Foxp3 expression in the spleen and mLN of host Rag1 KO mice. Consistent with Treg functional loss, Foxp3 expression was downregulated in PD-1H KO Treg (11% in spleen and 39% in mLN) compared with WT Treg (17% in spleen and 54% in mLN) (Supplementary Fig. S7D and E). However, absolute numbers of Foxp3^+^ T cells were insignificant between PD-1H KO Treg vs. WT Treg co-transferred mice (Supplementary Fig. S7F). Furthermore, there were significantly more IFN-γ^+^ cells in the mice transferred with PD-1H KO Treg (30.7% in spleen and 14.9% in mLN) vs. WT Treg (7% in spleen and 3.95% in mLN) (Supplementary Fig. S7G). Finally, more IL-17^+^ T cells appeared upon the transfer of PD-1H KO Treg (13.3% in spleen and 9.8% in mLN) than the WT Treg (3.64% in spleen and 4.44% in mLN) (Supplementary Fig. S7G and H). No difference was observed between IFN-γ^+^ and IL-17^+^ Teff (CD45.1) in the spleen and mLN after the transfer of PD-1H KO Treg/Teff vs. WT Treg/Teff (Supplementary Fig. S7I). Our results indicate that a lack of PD-1H on Treg leads to rapid conversion of Treg to other effector CD4 subsets which may facilitate inflammation. Therefore, PD-1H is required to maintain suppressive functions and Foxp3 expression of Treg cells under inflammation.

### Increased STAT3 activity and Foxp3 enhancer methylation in PD-1HKO iTreg cells

**Figure 5:**
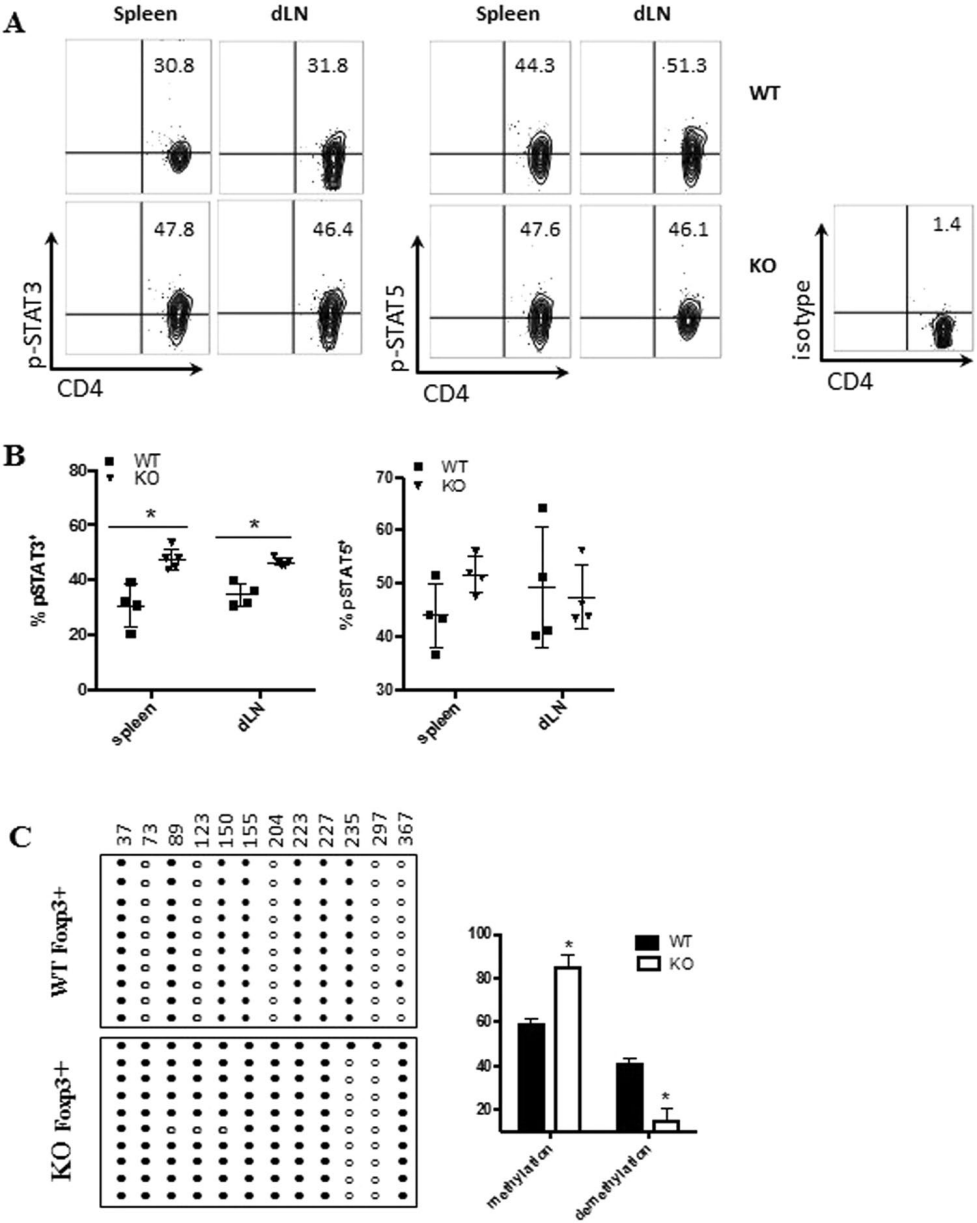
PD-1H promotes the commitment of iTreg cells. (**A**) The transferred iTreg cells from each group (gate on CD45.2^+^CD4^+^) in the EAE model were analysed on Day 13. The spleen and dLN cells were intracellularly stained with pSTAT3 or pSTAT5 with specific mAb. (**B**) Same as A but phosphorylation of STAT3 and STAT5 on transferred iTreg cells is displayed as plotted value. (**C**) The DNA methylation status of the CNS2 region was determined by bisulfite sequencing. Each line represents one clone (one DNA strand); open cycle, un-methylated cites; filled circles, methylated cites.

STAT5 activation drives Treg lineage commitment, whereas STAT3 is inhibitory for Foxp3 expression and promotes Th17 cell response. To examine whether the loss of PD-1H shapes the STAT pathways in Treg cells, we analysed the activation of STAT-5 and STAT-3 in the EAE model. PD-1H KO iTreg cells (up to 47% p-STAT3) expressed significantly higher levels of phosphorylated STAT-3 (p-STAT3) than WT iTreg cells in the spleen and dLN of EAE mice. However, comparable levels of p-STAT5 were found in both WT and PD-1H KO iTreg cells (Fig. 5A and B), implicating a role for STAT3 but not STAT5 in PD-1H function.

Previous studies showed that the DNA methylation status of the CNS2 (conserved noncoding DNA sequence 2) region of the Foxp3 enhancer was particularly important for maintaining Foxp3 expression. Here, we determined that methylation of the CNS2 region is in PD-1H KO iTreg cells. As shown in Fig. 5C, *in vitro* induced WT iTreg showed almost half as much demethylation of the CNS2 region (40.8% on average). However, PD-1H KO iTreg cells showed significantly less demethylation (17.5% on average), and hypermethylation of the CNS2 region of Foxp3 enhancer was 82.5% on average. This observation explains the instability of PD-1H KO iTreg cells on Foxp3 expression. Altogether, our data demonstrate that PD-1H deficiency has a significant impact on Treg’s differentiation and phenotype stability.

## Discussion

The results presented here show that PD-1H is a critical cell surface signalling molecule that controls the pool size of inducible Treg via two different mechanisms. First, PD-1H is required for the generation of iTreg from naïve T cells. This effect is largely mediated via suppression of inflammatory cytokine such as IFN-γ, IL-4, and IL-17. As a result, the pool size of iTreg that respond to environmental stimulation decreases. Although PD-1H does not affect the suppressive function of iTreg on a per cell level, the decreased pool size of iTreg may eventually affect the overall suppressive function of Treg during immune responses. Second, PD-1H signalling prevents the conversion of iTreg to Th1 and Th17 in an inflammatory environment. This effect may be due to the direct regulatory role of PD-1H on Foxp3 expression on iTreg cells. By promoting the generation of iTreg and preventing conversion of iTreg to Th1 and Th17, PD-1H helps to keep a constant level of iTreg during inflammation. To the best of our knowledge, this is the first comprehensive study that describes the mechanisms that underlie PD-1H’s mediation and mechanisms that control regulatory T cells.

While the coinhibitory function of PD-1H has been demonstrated in early studies^21, 23, 26^, the mechanisms underlying this effect have yet to be elucidated. Wang and colleagues showed that PD-1HIg partially promoted the differentiation of iTreg cells in the presence of TGF-β, and that this effect could be found in both mouse and human CD4^+^ T cells^28, 29^. In addition, a mAb to PD-1H reduced the differentiation of tumour antigen-specific iTreg cells *in vivo*^28^. In these studies, PD-1H is believed to be a ligand that engages a yet unidentified inhibitory receptor on Treg to mediate this effect. Our results using PD-1H deficient mice and PD-1H agonistic mAb showed that PD-1H acts as a co-inhibitory receptor on T cells^26, 30^. It should also be noted that the role of PD-1H in the suppression of immune responses may be more complex and may work through more than one single mechanism. Our recent studies demonstrate that PD-1H-mediated suppression of T cell tolerance to allogeneic antigens is mediated by two distinct mechanisms: an early arrest of cell proliferation and a late induction of Treg to maintain graft tolerance^30^. Here, we reveal a critical role for PD-1H in the control of Treg pool size which may contribute to the induction of long-term tolerance of T cell responses.

The effect of PD-1H on Treg is a highly selective event. Although PD-1H is essential for the generation of iTreg from naïve T cells, the suppressive function of both nTreg and iTreg is not affected. Notably, PD-1H is not required for the generation and suppressive function of nTreg cells, which are thymus-derived and selected by self-antigens (Supplementary Fig. S3). This result may partially explain why spontaneous autoimmune diseases or lymphoproliferative symptoms were not observed in young PD-1H KO mice. Nevertheless, spontaneously generated iTreg cells in naïve mice were clearly affected (Fig. 2A), which were previously described in activated CD4^+^ T cells in the presence of TGF-β and retinoic acid, and this population was mainly accumulated in the gut and skin mucosa^38^. Although there was a reduced frequency of Foxp3^+^ iTreg cells found in the intestine, especially in the LP of PD-1H KO mice, the absolute numbers of Foxp3^+^ iTreg in LP did not change (Fig. 2A). This may partially explain why PD-1H KO mice do not develop spontaneous pathogenic diseases in the gut. We also validated this finding in PD-1H KO mice that were bred with Foxp3 (GFP) mice (where GFP expression was under the control of a Foxp3 promoter) to generate PD-1HKO Foxp3 (GFP) mice (data not shown). These findings were further confirmed using an oral tolerance model for *de novo* generation of iTreg cells *in vivo* as previously described^31^. Consistent with the findings in the gut of PD-1H KO mice, PD-1H deficiency led to impaired *de novo* generation of antigen specific Foxp3^+^ iTreg cells in both mLN and PP (Fig. 1A and B). In addition, conversion of naïve CD4^+^ T cells into iTreg cells *in vitro* in the presence of TGF-β further proved that PD-1H deficiency reduced differentiation of iTreg cells (Fig. 2C). Finally, PD-1H deficiency in iTreg cells showed comparable suppressive functions when compared with WT iTreg cells, although minor differences could be found when the Treg/Teff ratio was high (Fig. 2D and E). Besides, we showed that, in the lymphopenic environment, loss of PD-1H on CD4^+^ T cells led to reduced numbers of Foxp3^+^ Treg in peripheral organs after homeostatic proliferation, while producing large quality of pro-inflammatory cells which further impeded the differentiation of Treg *in vivo* (Supplementary Fig. S2). A similar finding was also observed in the bone marrow of chimeric mice, the loss of PD-1H in hematopoietic derived cells led to a higher recovery of myeloid cells such as neutrophil, macrophage (data not shown), and the production of pro-inflammatory cytokines thus may impede the pool size of Treg cells in the periphery (Supplementary Fig. S4). Thus, our findings support a highly selective role for PD-1H in the generation of iTreg.

Treg is not terminally differentiated and it has been shown that iTreg can be converted into Th1 or Th17 subsets by a specific cytokine or inflammatory environment^39–43^. This plasticity of Treg cells makes it difficult to develop therapeutic strategies during chronic inflammation. Our data demonstrate that PD-1H is required for the stability of Foxp3, a hallmark of Treg lineage, and maintains the phenotype of Treg. Loss of PD-1H led to a rapid decrease of iTreg in various *in vitro* and *in vivo* systems and models. Our preliminary studies indicate that iTreg cells in the absence of PD-1H tend to be reprogrammed into Th17 cells in inflammatory conditions, which may explain our observations that transfer of PD-1H KO iTreg did not suppress but promoted EAE disease. In this model, conversion of iTreg was predominantly biased to Th17 but much less to Th1. PD-1H may also affect the conversion of iTreg to Th1 because high levels of IFN-γ were detected in CD4^+^ T cells in the absence of PD-1H^26^. Interestingly, our results showed that IL-4 levels were also significantly increased and these data implicate a possible role for PD-1H in the control of Th2. The role of PD-1H in the regulation and conversion of iTreg to other T cell subsets has yet to be elucidated.

Altogether, our data support that PD-1H inhibits the conversion of iTreg cells into Th1 and Th17 cells in an inflammatory environment, which is, at least partially due to its role in the maintenance of Foxp3 expression and an iTreg phenotype. Our findings have important implications in the regulation of Treg growth and function. Meanwhile, PD-1H inhibits activation of naïve T cells to limit initiation of T cell-mediated immune responses; as previously shown, it promotes growth and conversion of iTreg during immune responses. In addition to regulating early stage T cell activation, PD-1H appears to participate in the regulation of T cell tolerance by regulating Treg pool size. Thus the PD-1H pathway may represent a promising target to control and manipulate T cell-mediated immunity in inflammation, autoimmune disease, and cancer.

## Methods

### Mice

Eight-week-old C57BL/6 (B6) mice were purchased from Sun Yat-sen University Animal Supply Centre. PD-1H-KO mice on a B6 background were described previously^26^. The transgenic strain CD45.1, OT-II, Foxp3 (GFP), Rag1 KO were all purchased from Jackson Laboratory. Littermate mice of PD-1H KO and wild type (control mice) were generated from PD-1H heterozygotes and maintained in the same conditions. Foxp3 (GFP) mice and OT-II mice were backcrossed to the PD-1H KO mice respectively to generate PD-1H deficient Foxp3 (GFP) reporter mice and PD-1H deficient OT-II mice. Mice were maintained in a specific pathogen-free facility, and all animal experiments were performed in accordance with the National Institutes of Health Guide for the Care and Use of Laboratory Animals, with the approval of the Scientific Investigation Board of Sun Yat-sen University (Guangdong, China).

### Antibodies, kits and flow cytometry analysis

For cell surface staining of mouse PD-1H, MH5A (hamster anti-mouse PD-1H) was used^23^, followed by anti-hamster-IgG-PE (eBioscience); hamster IgG (eBioscience) was used as the isotype control. PD-1H agonist mAb clone mam82 (mouse anti-mouse IgG1) were described previously^26^. All other fluorescently labelled antibodies including CD4, CD25, CD44, CD69, Foxp3, TCR Vβ5.1/5.2, p-STAT3, p-STAT5, CTLA-4, Lag-3, GITR, IOCS, CD45.1, and CD45.2 were purchased from eBioscience and BD Pharmingen. For neutralizing assays, the anti-IFN-γ (clone XMG1.2), anti-IL-4 (clone 11B11), anti-IL-6 (clone MP5–20F3) neutralizing Abs were purchased from R&D System. The intracellular staining for Foxp3 and other intracellular cytokines were performed according to BD’s Cytofix/Cytoperm kit manual. Cytokine analysis was performed using the mouse Th1/Th2/Th17 CBA kits (BD Bioscience). Mouse pan-T isolation kit, CD8^+^ T cell isolation kit, CD4^+^ T cell isolation kit, CD25 micro beads kit, and naïve CD4^+^ T cell isolation kits were purchased from Miltenyi Biotec (Cambridge, MA). Flow cytometry analysis was performed using a BD FACSVerse (BD Biosciences) and the data was analysed using FlowJo software (Tree Star).

### Cell sorting and purification

Single cell suspension of spleen and LN were collected, and naïve CD4^+^CD25^−^CD44^lo^CD62^hi^ T cells were isolated using a naïve CD4^+^ T cell isolation kit (Miltenyi Biotec). For the Foxp3 (GFP) knock-in mice, CD4^+^CD25^−^Foxp3 (GFP^-^) T cells and CD4^+^CD25^+^Foxp3 (GFP^+^) nTreg were sorted by FACSAria (BD Biosciences) after purification using a CD4^+^ T cell isolation kit. In the indicated experiments, the CD4^+^ T cells were first enriched using a CD4^+^ T cell isolation kit, and CD25^+^ T cells were subsequently depleted using a CD25 microbead kit (Miltenyi Biotec) to obtain CD4^+^CD25^−^ T cells. The purity of cells sorted using this method was typically more than 95%.

### *In vitro* conversion of iTreg cells

The sorted CD4^+^CD25^−^Foxp3 (GFP^−^) T cells were stimulated *in vitro* with plate-bound anti-CD3 (clone 2C11 at 2 µg/ml, eBioscience) plus soluble anti-CD28 (1 µg/ml) in the presence or absence of recombinant TGF-β (5 ng/ml, R&D Systems) and IL-2 (5 ng/ml, PeproTech) for 3–5 days. Conversion of Foxp3^+^ Treg cells were then analysed by flow cytometry based on the expression of GFP or intracellular staining for Foxp3. In the indicated experiments, cells were cultured in plates coated with PD-1H agonist mam82 (10 µg/ml) after plate-coated with anti-CD3 (1 µg/ml). For the cytokine neutralization experiments, the mAb to IL-4, IL-6, and IFN-γ at 10 µg/ml were added to the wells at the beginning of the cultures. The cultured supernatants were collected at the indicated time points for the cytokine analysis.

### Suppressive assay of Treg cells

Treg cell suppressive functional assays were performed, as previously described^44^. Briefly, naïve CD8^+^ T cells (some experiments used the CD4^+^ naïve T cells) were first labelled with 1 μM CFSE (Life Technologies) and subsequently co-cultured at 1 × 10^5^ in the presence of 1 × 10^5^ mitomycin C-treated syngeneic spleen cells as feeder cells. The cultures were added with or without Treg cells at the indicated ratios in U-bottomed 96-well plates for 72 h; anti-mouse CD3 (1 μg/mL) was subsequently added for stimulation. Proliferations of T cells were assayed by CFSE dilution. In some experiments, soluble mouse control IgG or PD-1H agonist mam82 (10 μg/ml) was added to the cultures. In these cases, the feeder cells were the PD-1H KO spleen cells.

### Oral tolerance mouse model

*De novo* generation of Foxp3^+^ iTreg cells were performed according to published protocols^31^. Briefly, naïve CD4^+^CD25^−^ T cells were isolated from OT-II mice (or PD-1HKO OT-II mice) as previously described and labelled with 5 μM CFSE before adoptive transfer. Typically, 2 × 10^6^ cells were injected i.v. into WT B6 mice. After 24 hours and for 5 consecutive days, the drinking water in the cages was replaced with a 1.5% OVA solution (grade V; Sigma-Aldrich). On day 6, the mesenteric LN and PP were collected and the TCR-specific Foxp3^+^ Treg cells were determined using intracellular staining for Foxp3 by flow cytometry.

### Bone marrow chimeras

Bone marrows from the tibia and femurs of WT(CD45.1) and PD-1H KO mice (CD45.2) were mixed at a 1:1 ratio and a total 1 × 10^7^ cells were transferred into sub-lethal irradiated (6Gy) congenic WT mice (CD45.1/CD45.2). The indicated organs were analysed 10 weeks later, after reconstruction. The prevalence of Foxp3^+^ cells was determined by intracellular staining.

### EAE disease model

The experimental autoimmune encephalomyelitis (EAE) model was used as previously described^45^. Briefly, 7-8 week old female mice were immunized s.c. with 200 μg MOG_35–55_ (Life Technologies) in complete Freund’s adjuvant (Difco). Mice were given 400 ng pertussis toxin (List Biological Labs) in 500 μl PBS i.p. on day 0 and 2 post-immunization. For adoptive transfer, 1 × 10^6^ WT Foxp3 (GFP^+^) iTreg cells or PD-1HKO Foxp3 (GFP^+^) iTreg cells were injected i.v. before immunization on day 0. The same volume of PBS (saline) was injected i.v. into WT mice and served as a control. Mice were observed daily and clinical scores were determined (in a blinded manner) on a scale of 0-5, as follows: 0 = healthy; 1 = limp tail; 2 = limp tail and hind limp weakness; 3 = hind limp paralysis; 4 = all hind limps paralysis and forelimb weakness; 5 = moribund condition. For an *ex vivo* analysis of cells obtained from EAE mice, the spleens and dLN from each group were collected, and cell suspensions were re-stimulated with PMA/Ionomycin/BD GolgiPlug for an additional 4 hours. The level of IFN-γ and IL-17 was assessed using intracellular staining and analysed by FACS (BD Cytofix/Cytoperm kit).

### T cell transfer model of chronic colitis

Briefly, Foxp3^+^ iTreg cells from WT and PD-1H KO mice were prepared and sorted as described above. Syngeneic CD4^+^CD45RB^hi^ T cells (CD45.1, 4 × 10^5^) were co-injected via i.p. with or without 2 × 10^5^ iTreg cells (CD45.2) into Rag1 KO mice. Mice were weighed once a week. After 10 weeks, the colon tissue was collected and histologically stained with H&E. Spleen and mLN cells were collected and *ex vivo* activated using PMA/Ionomycin/BFA for 4 hours, and then the cytokine production was analysed.

### DNA Methylation analysis

The Foxp3 (GFP^+^) iTreg cells were sorted, and genomic DNA was purified using a DNeasy Blood & Tissue Kit (Qiagen). The bisulfite conversion of DNA was performed using EZ DNA Methylation-Gold Kit (ZYMO research). The CNS2 region of the Foxp3 enhancer was amplified using a primer set, as previously described^46^, and T/A cloned into pMD18-T vector (Clonetech). Ten inserted plasmids from each group were purified and sequenced and the methylation results were analysed using BiQ Analyzer 2.0.

### Graphs and statistical analysis

The graphs and data analyses were generated using Graph Pad Software. Statistical analyses were performed using unpaired student’s t tests, *P* values of less than 0.05 were considered significant. Error bars in figures represent standard error (SE). For the disease progression, the two-way ANOVA test (*p < 0.05) was used. All experiments were repeated at least 3 times.

## Electronic supplementary material

Supplementary Figures
