# Author Correction: Programmed death one homolog maintains the pool size of regulatory T cells by promoting their differentiation and stability

**DOI:** 10.1038/s41598-023-37791-w

**Published:** 2023-07-18

**Authors:** Qi Wang, Jianwei He, Dallas B. Flies, Liqun Luo, Lieping Chen

**Affiliations:** 1 Laboratory of Immunotherapy, Sun Yat-Sen University, Guangzhou, Guangdong P.R. China; 2 Department of Immunobiology, Yale University, New Haven, Connecticut USA

Correction to: *Scientific Reports* 10.1038/s41598-017-06410-w, published online 20 July 2017

This Article contains an error in the Acknowledgements section.

“We thank Beth Cadugan for editing the manuscript, Dr. Zhiyong Guo (The first affiliated hospital, Sun Yat-sen University) for generously sharing the Foxp3(GFP) knock-in mice, and Mr. Xiaobo Li for providing the technical support of cell sorting. This study partially fulfills the requirement of the Ph.D. degree at the Graduate School of Sun Yat-sen University School of Medicine for Qi Wang and Jianwei He. This work is supported in part by the 985 project grant from Sun Yat-sen University, Guangdong Province Innovative Research Program Project 2011Y035, PRC; National Institutes of Health grants P50 CA177444, P30 CA016359, P50 CA17744415 and P30 CAO16359, and an endowment from the United Technologies Corporation, USA.”

should read:

“We thank Beth Cadugan for editing the manuscript, Dr. Zhiyong Guo (The first affiliated hospital, Sun Yat-sen University) for generously sharing the Foxp3(GFP) knock-in mice, and Mr. Xiaobo Li for providing the technical support of cell sorting. This study partially fulfills the requirement of the PhD degree at the Graduate School of Sun Yat-sen University School of Medicine for Qi Wang and Jianwei He. This work is supported in part by the 985-project grant from Sun Yat-sen University, Guangdong Province Innovative Research Program Project 2011Y035, PRC; National Institutes of Health grants P50 CA177444, P30 CA016359, and an endowment from the United Technologies Corporation, USA.”

