# Supplementary material for: Programmed death one homolog maintains the pool size of regulatory T cells by promoting their differentiation and stability
Source: Sci Rep. 2017 Jul 20;7:6086. doi: 10.1038/s41598-017-06410-w (PMC5519767; doi:10.1038/s41598-017-06410-w)

Supplemental Materials

Programmed death one homolog maintains the pool size of regulatory T cells by promoting their differentiation and stability

Qi Wang[1], Jianwei He[1], Dallas B. Flies[2], Liqun Luo[1] and Lieping Chen[1,2]

[1]Laboratory of Immunotherapy, Sun Yat-Sen University, Guangzhou, Guangdong, PRC
[2]Department of Immunobiology, Yale University, New Haven, Connecticut, USA

Correspondence: Lieping.Chen@yale.edu

Supplementary Figure 1: PD-1H affects *de novo* differentiation of iTreg cells. (A) WT and KO OT-II T cells ($CD25^-$ T cells) were transferred into host mice separately. The transferred OT-II T cells were labelled with CFSE before being transferred. The figure shown here is the dilution of CFSE and Foxp3 induction. (B) WT (CD45.1/CD45.2) naïve OT-II T cells and KO (CD45.2) OT-II naive T cells were mixed at a 1:1 ratio and co-transferred into host mice (CD45.1). After orally feeding the host mice with 1.5% OVA, the mLN and PP were analysed and the frequency of Foxp3 were determined by intracellular staining. (C) The absolute number of $Foxp3^+$ T cells in the indicate organs were counted.

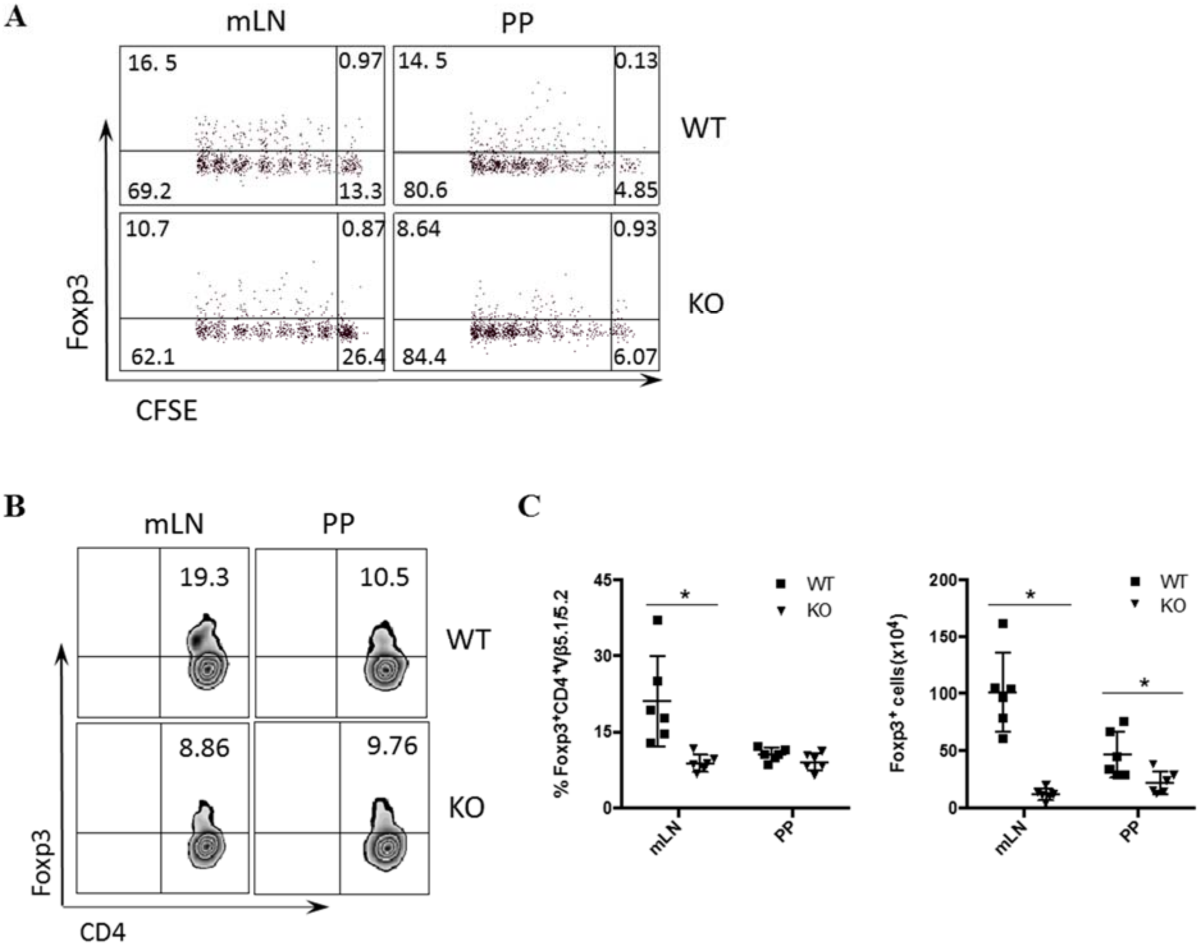

Supplementary Figure 2: PD-1H regulates differentiation of iTreg cells in a lymphopenic environment. (A) Naïve $CD4^+CD62L^{hi}$ T cells from WT mice (CD45.2) and PD-1H KO mice (CD45.1) were mixed at ratio of 1:1 and a total of 2 million cells were transferred into Rag1 KO mice (n=4); Foxp3's upregulation was determined 20 days later. This figure shows the change in ratio of WT and KO $CD4^+$ T cells before and after transfer. The frequency of $Foxp3^+$ cells was determined by intracellular staining. (B) Absolute numbers of $CD25^+Foxp3^+$ T cells in the indicated organs from the Rag1 KO mice were counted. (C) Cytokine production of transferred naïve $CD4^+$ T cells was analysed. Cells from the indicated organs were activated *in vitro* for 4 hours in the presence of PMA/Ionomycin/BFA and then analysed by intracellular staining. Data shown are representative of 2 independent experiments.

.

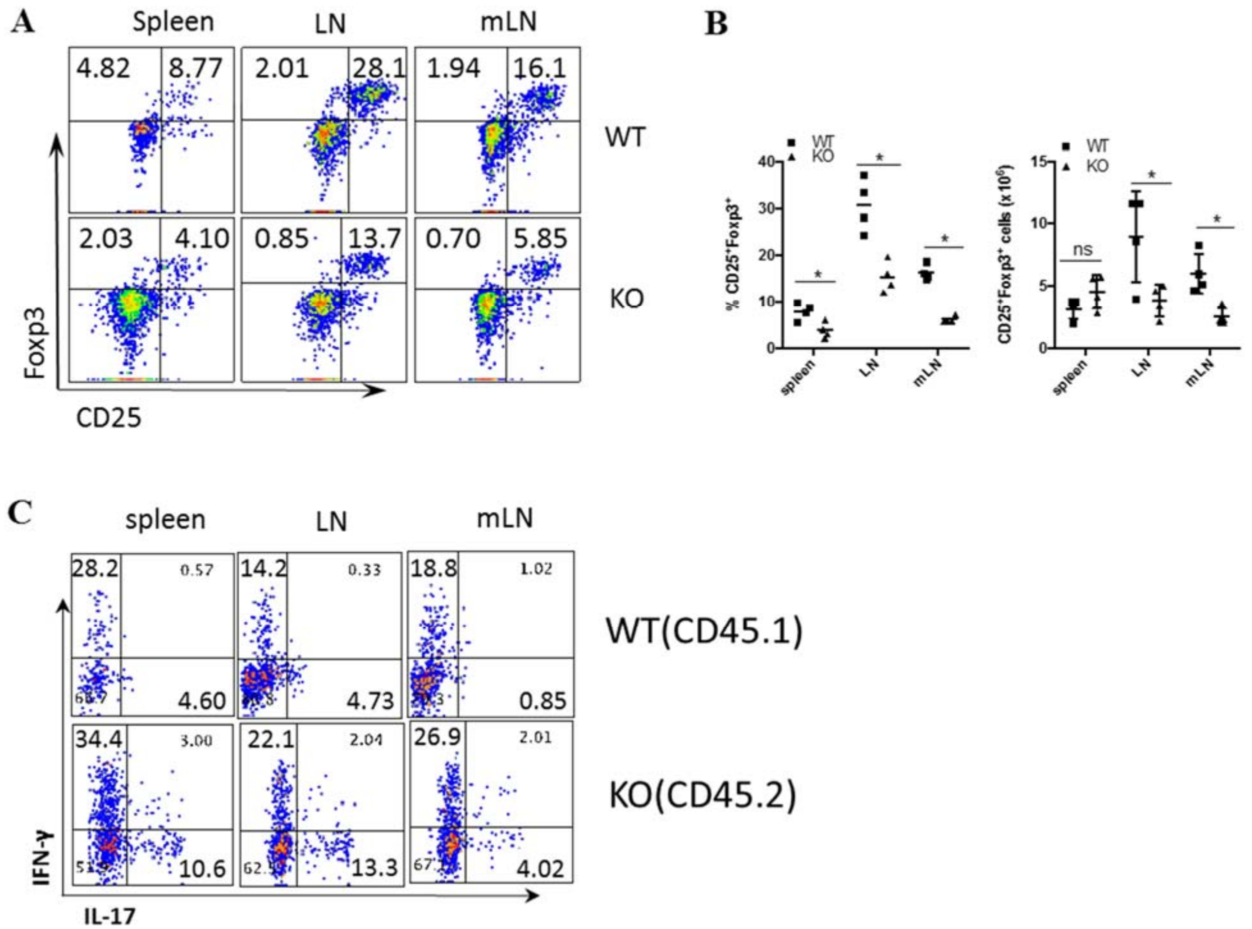

Supplementary Figure 3: PD-1H plays a redundant role on the generation and suppressive function of nTreg cells. (A) The percentage of $CD25^{+}Foxp3^{+}$ nTreg in the spleen and LN was determined by Flow cytometry using intracellular staining for Foxp3 and cell surface staining of CD25. Six weeks old littermates from WT and PD-1H KO mice (n=5) were used. The absolute numbers of $Foxp3^{+}CD25^{+}$ T cells in the spleen and LN were counted. (B) The expression of CD25, GITR, Lag-3, CTLA-4, ICOS and PD-1 on the nTreg from WT or PD-1H KO Foxp3(GFP) mice were determined by flow cytometry gating on the $CD4^{+}$Foxp3(GFP^{+}) cells. (C) Foxp3(GFP^{+}) nTreg from WT or PD-1H KO Foxp3(GFP) mice were sorted and co-cultured at various Treg/Teff ratios with CFSE-labelled $CD8^{+}$ Teff cells in the presence of mitomycin C treated spleen cells plus anti-CD3 for 3 days. The CFSE dilution was determined by flow cytometry. Data shown are representative of at least 3 independent experiments.

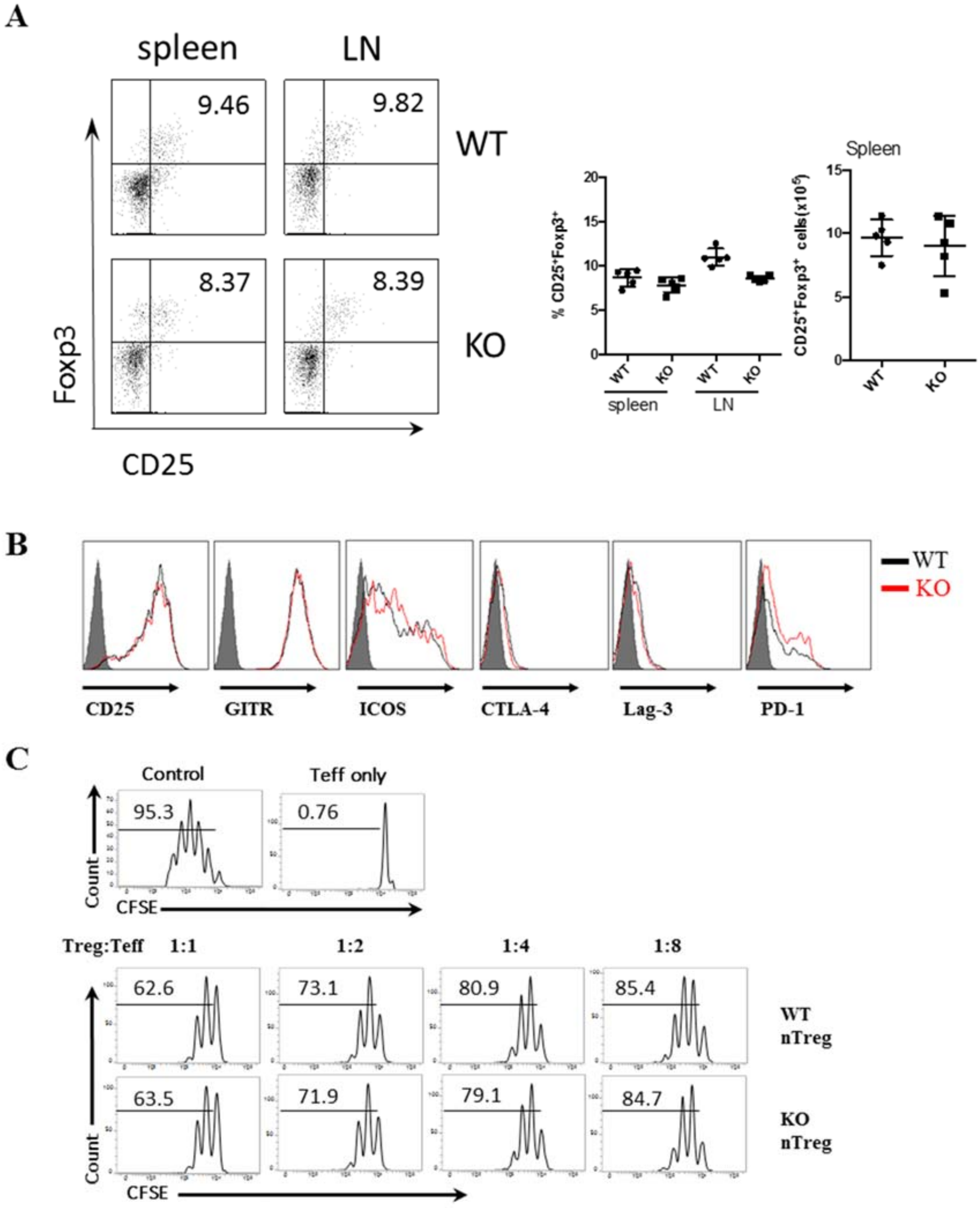

Supplementary Figure 4: PD-1H determines the pool size of Treg cells in bone marrow chimeric mice. (A) A total of 10 million mixed bone marrow cells from CD45.1 WT mice and CD45.2 mice were transferred into sub-lethally irradiated mice (CD45.1/CD45.2). Mice were analysed 10 weeks after reconstruction. The gating strategy for the WT and KO T cells was shown. (B) Foxp3 frequency in the indicated organs was determined by intracellular staining. (C) Foxp3 frequency in the spleen were summarized, and the absolute number of Foxp3^{+} cell and Foxp3^{-} cells were shown. Data shown are one of two independent experiments.

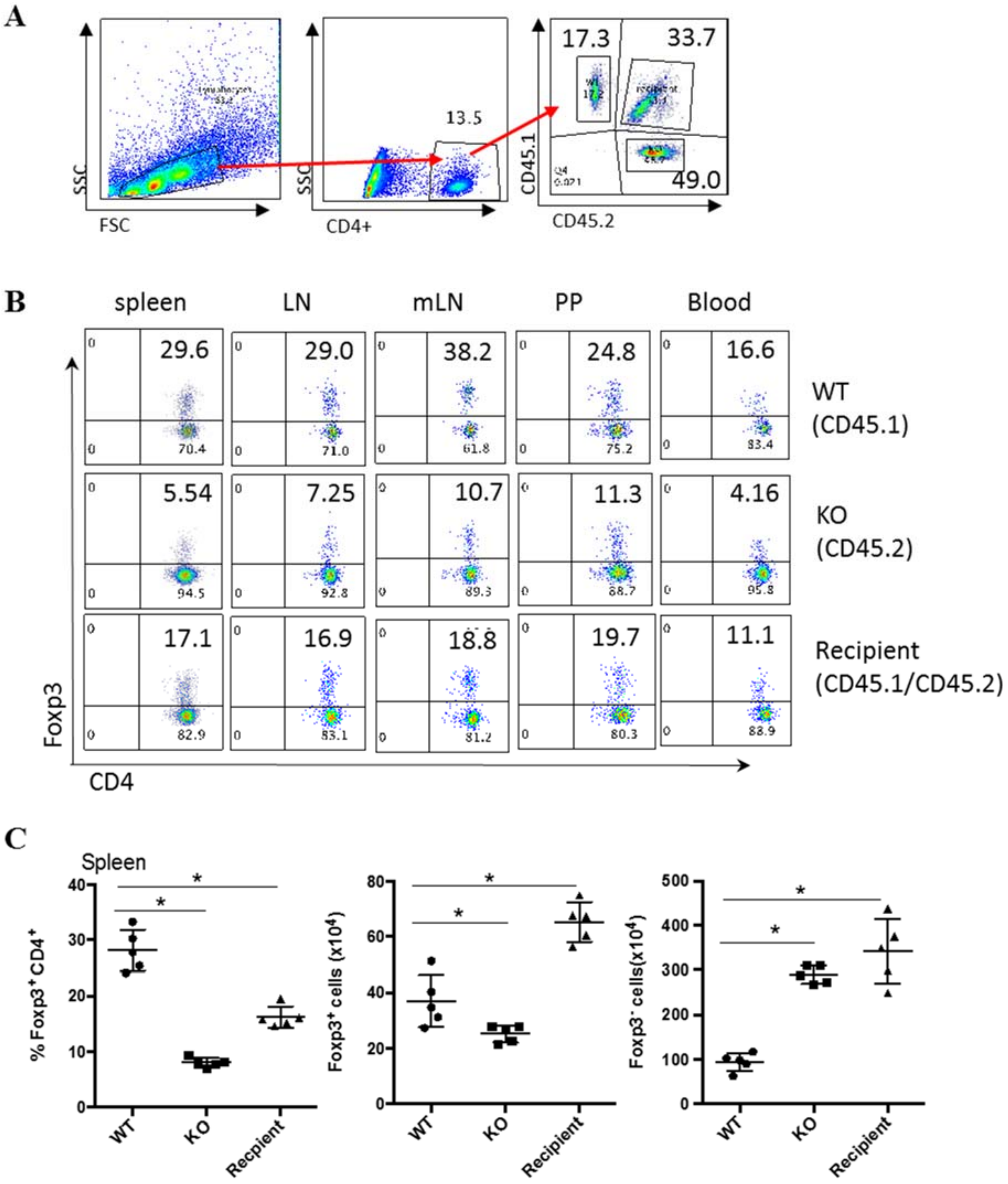

Supplementary Figure 5: PD-1H agonist mAb slightly promotes the differentiation of iTreg cells (A) Naïve T cells were sorted from the WT Foxp3 (GFP) mice and subsequently stimulated with pre-coated anti-CD3 with either control mouse IgG or mam82 in the presence of TGF-β for 4 days. $CD25^{+}$Foxp3 (GFP^{+}) iTreg cells were analysed by flow cytometry. (B) Induction of iTreg cells at different time points were shown. The results shown are from 3 individual experiments. Data shown are representative of at least 3 independent experiments.

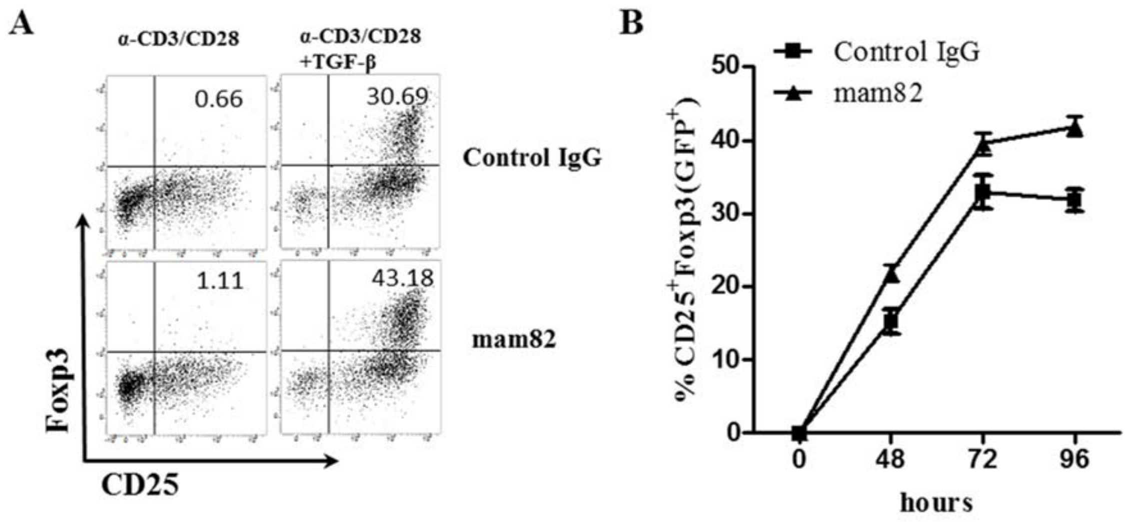

Supplementary Figure 6: Analysis of CD4^{+} Th1 and Th17 cells in recipients upon transferring with WT or PD-1H KO iTreg cells in the EAE model. (A) Naïve T cells from WT or PD-1H KO Foxp3 (GFP) mice were differentiated into iTreg cells *in vitro* as described above. IFN-γ and IL-4 neutralizing mAb were added to the cultures to promote the generation of iTreg. Foxp3 (GFP^{+}) cells were sorted and the purity was assessed by FACS. (B,C) In the EAE model after the transfer of iTreg cells, recipient CD45.1^{+}CD4^{+} T cells were re-stimulated *ex vivo* by PMA/Ionomycin/BFA. IFN-γ^{+} or IL-17^{+} cells of recipient CD4^{+} T cells were determined using intracellular staining, gated on CD45.2^{-}CD4^{+} T cells. Data from a representative pair of mice were shown in (B) and a graphic plot of the data was shown from a group of 4 or 5 mice (C). Data shown were representative of at least 3 independent experiments.

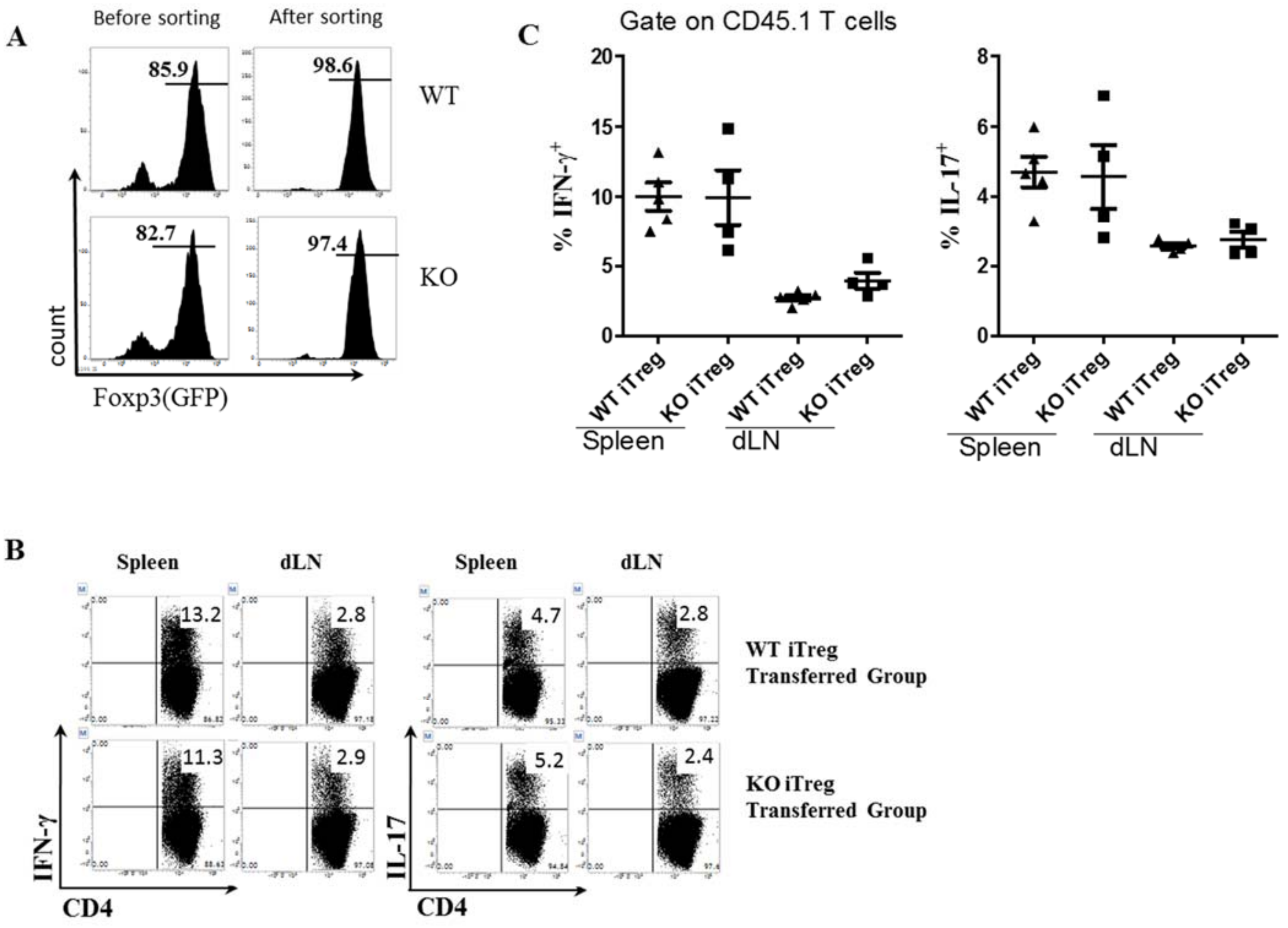

Supplementary Figure 7: PD-1H deficient iTreg cells fail to retain their suppressive function and Foxp3 expression. (A) The weight of Rag1 KO host mice (n=5 per group) at various times after the transfer of $CD25^{-}CD45RB^{hi}$ CD45.1 T cells alone or together with $CD45.2^{+}Foxp3(GFP^{+})$ iTreg cells from WT or KO mice (As described in Methods). Weight changes of recipient mice after transfers were normalized to their initial weight before the transfer. *$P<0.05$, (two-way ABOVA test). (B) H&E staining of colon tissues (Scale Bar: 100 μm) and clinical score (C) from the Rag1 KO host mice after inducing colitis (week 10, n=5). (D, E, F) Percentage of $Foxp3^{+}$ T cells among CD45.2 Treg cells in the spleen and dLN from Rag1 KO host mice were analysed and absolute numbers of $Foxp3^{+}$CD45.2 T cells were counted based on the ratio and live cells. (G) Flow cytometry analysing the expression of IFN-γ and IL-17 in CD45.2 Treg cells in the indicated organs from Rag1 KO mice. Spleen cells and dLN cells were stimulated with PMA/Ionomycin/BFA *in vitro* for 4 hours and stained for intracellular cytokine. (H) Summary of data in (G), each dot represents one mouse. (I) Flow cytometry analysing the expression of IFN-γ and IL-17 in Teff cells (CD45.1) in the indicated organs from Rag1 KO host mice. Spleen cells and dLN cells were stimulated with PMA/Ionomycin/BFA *in vitro* for 4 hours and stained for intracellular cytokines. Data shown are representative one of 2 independent experiments.

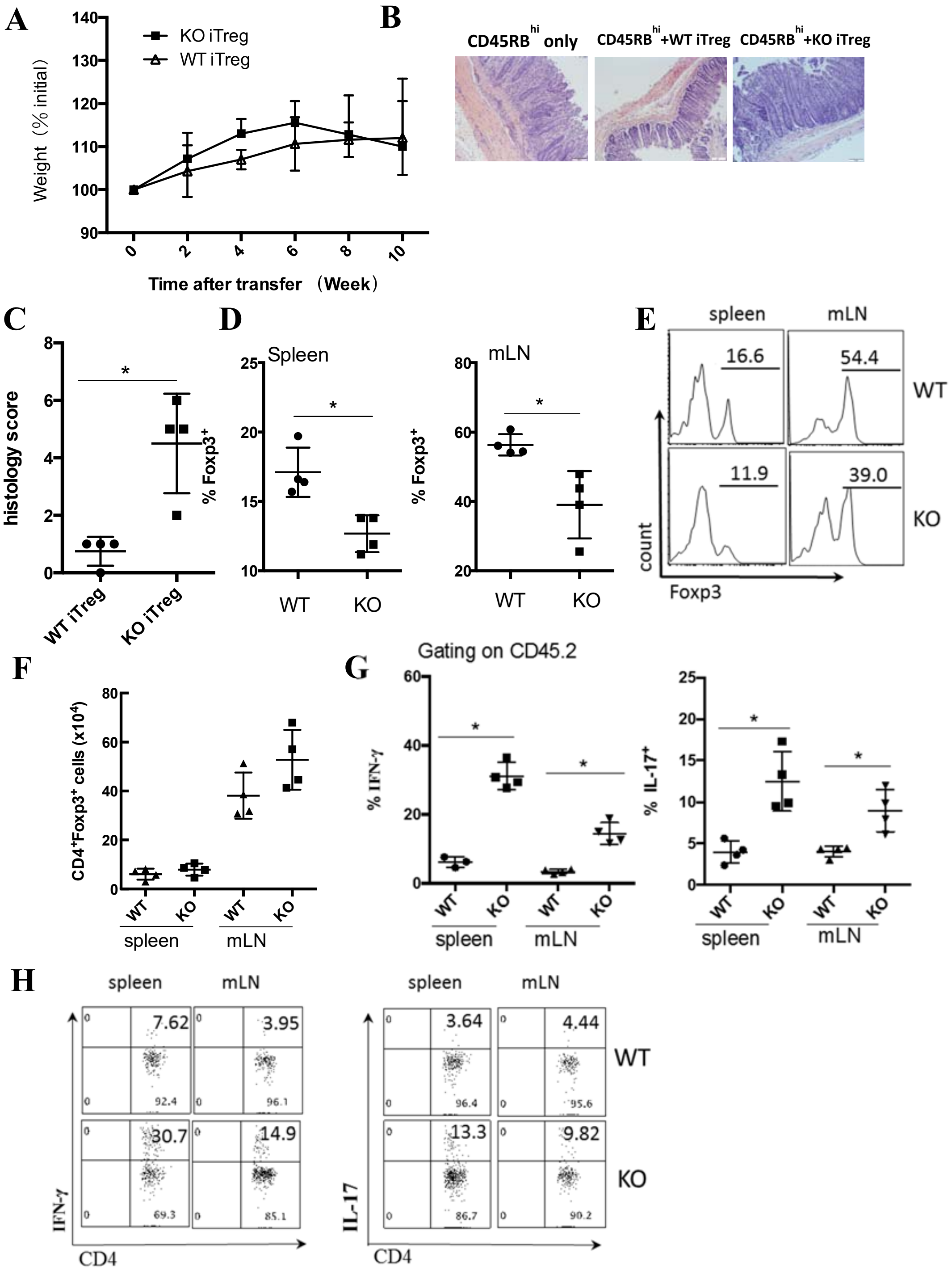

I Colitis model-activation of Teff (CD45.1)

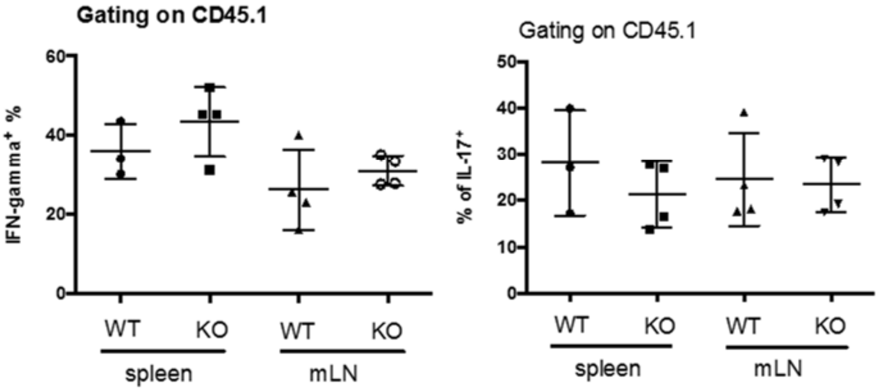

Supplement: Supplementary file 1 — Supplementary Figures [file 41598_2017_6410_MOESM1_ESM.pdf]
